# Hydroelectric Plant Safety: Real-Time Monitoring Utilizing Fiber-Optic Sensors

**DOI:** 10.3390/s24144601

**Published:** 2024-07-16

**Authors:** Renato Luiz Faraco, Felipe Barino, Deivid Campos, Guilherme Sampaio, Leonardo Honório, André Marcato, Alexandre Bessa dos Santos, Clayton Cesar dos Santos, Fernando Hamaji

**Affiliations:** 1 Instrumentation and Telemetry Laboratory, Federal University of Juiz de Fora, Juiz de Fora 36036-900, Brazil; 2 Departamento de Energia Elétrica, Federal University of Juiz de Fora, Juiz de Fora 36036-900, Brazil; 3 Santo Antônio Energia, Porto Velho 04548-001, Brazil

**Keywords:** fiber Bragg gratings, structural health monitoring, optical sensors, deformation, hydraulic power plant

## Abstract

In the context of hydroelectric plants, this article emphasizes the imperative of robust monitoring strategies. The utilization of fiber-optic sensors (FOSs) emerges as a promising approach due to their efficient optical transmission, minimal signal attenuation, and resistance to electromagnetic interference. These optical sensors have demonstrated success in diverse structures, including bridges and nuclear plants, especially in challenging environments. This article culminates with the depiction of the development of an array of sensors featuring Fiber Bragg Gratings (FBGs). This array is designed to measure deformation and temperature in protective grids surrounding the turbines at the Santo Antônio Hydroelectric Plant. Implemented in a real-world scenario, the device identifies deformation peaks, indicative of water flow obstructions, thereby contributing significantly to the safety and operational efficiency of the plant.

## 1. Introduction

Hydroelectric power plays a critical role in global energy production, contributing to sustainable and renewable energy solutions. Ensuring the structural integrity and operational efficiency of hydroelectric plants is paramount, given their significant capacity and the potential consequences of structural failures. Advanced monitoring systems are essential to safeguard these structures, optimize performance, and prevent catastrophic failures.

### 1.1. Research Background

Structures such as bridges, power towers, railways, pavements, and buildings face continuous challenges arising from loads and environmental conditions, resulting in degradation and potential structural damage. The implementation of structural health monitoring (SHM) systems becomes essential to reinforce the safety and longevity of these constructions, allowing constant surveillance and uninterrupted monitoring of the structural condition.

In this context, the crucial importance of displacement and deformation sensors is emphasized, playing a fundamental role in providing precise data on structural integrity [1]. These sensors constitute key elements in SHM systems, enabling a detailed assessment of structural conditions and facilitating early detection of potential issues, thus contributing to the effectiveness of maintenance and preservation strategies. In systems of this scale, the crucial importance of displacement and deformation sensors is highlighted.

The majority of the literature on structural health monitoring (SHM) predominantly relies on resistive strain gauges [2,3,4,5,6]. However, it is important to note that this type of deformation sensor has some limitations, such as a reduced signal-to-noise ratio (SNR), significant signal attenuation, cross-sensitivity to high temperatures, and the need for multiple wires for power supply and signal transmission.

Although some alternatives aim to improve the SNR and reduce the need for wiring [7], these devices still rely on electrical signals, which are susceptible to electromagnetic interference and exhibit high attenuation in long-distance applications.

Addressing the scenario of hydroelectric plants, the importance of structural monitoring becomes even more crucial. Following a severe accident in 2009 at the Sayano–Shushenskaya Hydroelectric Power Plant (SS HPP), resulting in the failure of all 10 hydroelectric units with a total capacity of 6400 MW [8], the need to develop robust methodologies to monitor operation and ensure the safety of these facilities became evident. Subsequent studies highlighted the effectiveness of approaches based on monitoring the natural frequencies of dam structures and forced vibrations caused by equipment operation [9].

Furthermore, Hsu et al. [10] demonstrated a continuous and effective strategy for monitoring the structural health of dams. Additionally, the work of Selak et al. [11] stands out as it presented a Condition Monitoring and Fault Diagnosis (CMFD) system for hydroelectric plants.

The application of fiber-optic sensors (FOSs) is a promising approach in this context due to their ease of transmission through an optical connection, low signal attenuation, and immunity to electromagnetic interference. These characteristics make FOSs ideal for measurements near intense magnetic fields, such as transmission lines and large turbines used in electricity generation [12].

Fiber-optic sensors (FOSs) are a prominent example of progress in optical sensor technology. Optical sensors are particularly well suited for and have been successfully employed in remote sensing applications. These devices cover a varied spectrum of applications and have been successfully used for crack monitoring [13,14], bridges [15,16,17,18], and concrete structures [19,20,21,22].

However, it is important to note that FBG arrays, despite their ability to function as quasi-distributed sensors, lack the capacity to monitor various properties throughout a structure due to their limited distributed sensing capabilities. To address this limitation, various distributed sensors based on Optical Frequency Domain Reflectometry (OFDR) and Optical Time-Domain Reflectometry (OTDR) have been developed.

OFDR offers high spatial resolution and sensitivity, making it ideal for detecting small changes over short distances [23]. This technique can provide continuous strain or temperature profiles along the length of the fiber [24,25]. However, it is typically limited by the coherence length of the laser source and can be more complex and costly to implement.

On the other hand, OTDR is widely used for its simplicity and robustness. It can measure long distances and is less affected by the coherence length of the light source. While OTDR offers lower spatial resolution compared to OFDR, it is highly effective in identifying faults and losses in optical fibers, making it suitable for long-range applications [26,27,28]. OTDR technology has also been successfully applied in structural health monitoring, as demonstrated in [29].

The advantages of employing FBGs include their high sensitivity, ease of multiplexing, thermal stability, and ability to operate in harsh environments. Recent studies have demonstrated the effectiveness of FBGs in various applications, such as structural health monitoring and temperature sensing in challenging environments. Additionally, FBGs exhibit low cost, fast response, and the ability to perform precise strain and temperature measurements in composite materials and smart structures [30].

Moreover, optical sensors not only excel in conventional structural monitoring applications but also prove promising for challenging environments, such as nuclear power plants. Studies have demonstrated their effectiveness and resilience in nuclear conditions, establishing these devices as a robust and reliable option for monitoring systems in critical environments [31,32,33].

### 1.2. Research Contributions

Implementing optical sensors in the protective grid of generating turbines in hydroelectric plants emerges as a strategic measure. This work describes the development of a sensor array with Fiber Bragg Gratings (FBGs) to measure deformation and temperature in the protective grids of generating turbines at the Santo Antônio Hydroelectric Plant.

The Santo Antônio Hydroelectric Plant, located on the Madeira River in Porto Velho, Rondônia, comprises 50 bulb-type turbines, each generating approximately 71.6 MW, with a total installed capacity of 3568.3 MW, making it the fifth-largest operational hydroelectric plant in Brazil and one of the largest in the world.

The device developed in this study was utilized to identify deformation peaks, revealing the presence of materials such as logs, stones, or plants deposited on the protective grids. This allows for the assessment of potential obstructions to water flow, contributing significantly to the safety and operational efficiency of the plant.

Additionally, the methodology implemented in this study emphasizes the robustness and reliability of FBG sensors in harsh environments. By detailing the experimental setup, encapsulation techniques, and testing procedures, this work provides a comprehensive guide for future research in similar applications. The insights gained from the field tests at the Santo Antônio Hydroelectric Plant can inform the design and deployment of SHM systems in other hydroelectric facilities, ensuring their structural integrity and operational safety.

### 1.3. Future Research Directions

Building on the findings of this study, future research could explore the integration of FBG sensors with advanced data analytics and machine learning algorithms to enhance real-time monitoring and predictive maintenance capabilities. Additionally, investigating the long-term performance and durability of FBG sensors under various environmental conditions would further validate their applicability in SHM systems.

## 2. Materials and Methods

### 2.1. Operating Principles of Fiber Bragg Gratings

Fiber-optic sensors are extremely interesting due to their logistical and metrological advantages, such as low weight, low power consumption, electromagnetic immunity, high sensitivity, low signal attenuation, environmental robustness, and resistance [34]. Among these technologies, we can highlight diffraction gratings, including Fiber Bragg Gratings (FBGs), first introduced in [35].

The detection principle of an FBG is based on a periodic disturbance of the refractive index along the length of the fiber, induced by exposing the core to intense optical interference patterns [36]. In these sensors, light is coupled to a mode opposite to the main mode, acting as an extremely selective optical mirror, as the reflection occurs centered in a very narrow band of the optical spectrum. The most intense interaction or mode coupling occurs at the Bragg wavelength, given by Equation (1):

(1) $$\lambda_{B}=2n_{eff}\Lambda$$

where $\lambda_{B}$ represents the Bragg wavelength, $n_{eff}$ is the effective refractive index of the fiber core, and Λ denotes the grating period [37].

For a thermal sensor model, the temperature variation not only changes the refractive index of the FBG but also causes thermal expansion, which changes the grating pitch [38]. Thus, disregarding the waveguide effect, the thermal response characteristic of the FBG can be described according to Equation (2):

(2) $$\frac{\Delta \lambda_{B}}{\lambda_{B}}=\left(\alpha +\zeta \right)\Delta T$$

where $\Delta \lambda_{B}$ is the variation in the Bragg wavelength, α is the coefficient of thermal sensitivity, and ζ is the thermo-optic sensitivity coefficient of the fiber. We also have the FBG temperature configuration equation according to (3):

(3) $$T={\left(\lambda -\lambda_{0}\right)}^{2}\cdot S_{2}+\left(\lambda -\lambda_{0}\right)\cdot S_{1}+S_{0}$$

where λ is the measured wavelength in nm, $\lambda_{0}$ is the reference wavelength in nm, *T* is the temperature in °C, $S_{0}$ is the zero-order calibration factor in °C, $S_{1}$ is the first-order calibration factor in °C/nm, and $S_{2}$ is the second-order calibration factor in °C/nm.

However, typically, the FBG is deformed due to external load or temperature, resulting in a change in the grating period, followed by a shift in the Bragg wavelength [39]. Therefore, it is possible to determine the variation in the Bragg wavelength caused by temperature and strain effects through Equation (4). Here, *k* is the calibration factor of the Bragg grating, a dimensionless value that correlates the strain-induced wavelength variation with the Bragg wavelength of an FBG, and Δϵ is the variation in strain in microstrains.

(4) $$\frac{\Delta \lambda_{B}}{\lambda_{B}}=k\Delta \epsilon +\left(\alpha +\zeta \right)\Delta T$$

The deformation measured in a mechanically loaded material, which is also subject to a temperature change, is affected by the load ($\epsilon_{load}$) and the thermal expansion of the material ($\epsilon_{\alpha }$). Thus, the actual deformation of the material is given by $\epsilon_{real}=\epsilon_{load}+\epsilon_{\alpha }$. It is necessary to compensate for temperature, considering both thermal effects, for accurate deformation measurement. To achieve this, the temperature effects on the measurement must be taken into account, usually determined experimentally and provided by the manufacturer in the sensor documentation, as they may differ from the theoretical behavior of silica [40,41]. This relationship is expressed by the term Temperature-Cross Sensitivity (TCS), as shown in Equation (5):

(5) $$\frac{\Delta \lambda_{B}}{\lambda_{B}}\cdot \frac{10^{6}}{k}=\epsilon +\alpha \cdot \Delta T+TCS\cdot \Delta T$$

It is important to highlight that when using an array of sensors that includes an FBG specifically designed to measure temperature and not susceptible to mechanical deformations, it becomes feasible to perform temperature compensation in the strain sensors through this optical element—the temperature sensor. To achieve this, it is necessary to consider Equation (5) for both sensors. However, as ϵ=0 for the temperature sensor, in this case, we have Equation (6):

(6) $$\Delta T=\frac{\Delta \lambda_{B_{temp}}}{\lambda_{B_{temp}}}\cdot \frac{10^{6}}{k_{temp}}-\frac{1}{\alpha_{temp}+TCS_{temp}}$$

Therefore, it is possible to apply Equation (6) in the model of Equation (5). By isolating ϵ, we can obtain the strain value for the strain sensor after temperature compensation, as shown in Equation (7):

(7) $$\epsilon =\frac{\Delta \lambda_{B}}{\lambda_{B}}\cdot \frac{10^{6}}{k}-\frac{\Delta \lambda_{B_{temp}}}{\lambda_{B_{temp}}}\cdot \frac{10^{6}}{k_{temp}}\cdot \frac{\alpha +TCS}{\alpha_{temp}+TCS_{temp}}$$

### 2.2. Array Assembly

The development of a sensor array is a process that involves the proper selection of devices and the specific positioning of each sensor, as the appropriate coordination of multiple sensors enhances comprehensive data collection. In this context, two FBGs were used for strain measurement, and one FBG was employed for temperature measurement, aiming to perform temperature compensation for the strain sensors, as described in Equation (7).

The FBGs used were encapsulated in a rubber apparatus to protect the sensors and metal at their edges, allowing the sensors to be welded to the protective grid. Each FBG was manufactured from standard single-mode SMF-28 fibers. These FBGs feature a uniform grating structure fabricated using the ultraviolet writing process. In the case of the temperature sensor, its encapsulation, in addition to the rubber and metal apparatus, was designed so that the sensor grid remained suspended in a rigid tube, avoiding influences from deformation. It is important to note the specific calibration configurations of the FBGs used in our study. The temperature sensor FBG was calibrated with a central wavelength ($\lambda_{0}$) of 1544.88746nm, corresponding to a period of approximately 531.9384nm. Its calibration parameters included $S_{0}$ = 30 °C, $S_{1}$ = 34.8 °C/nm, and $S_{2}$ = −0.8 °C/nm. Additionally, the strain sensor FBGs had central wavelengths of 1550.32209nm and 1570.32871nm, with periods of approximately 533.8097nm and 540.6984nm, respectively. Both strain sensors were calibrated with k=0.76±0.03 and TCS=7.6±1.

The FBGs were deliberately positioned at intervals of 1 m from each other along the grid structure. This spacing ensured optimal coverage and measurement accuracy, with the temperature sensor FBG strategically located between the deformation sensor FBGs. Therefore, the spacing between consecutive FBGs was precisely 1 m. This configuration aimed to achieve two main objectives. Firstly, it aimed to compensate for the effects of deformation in the structure, using the temperature sensor as a central reference. This approach aimed to minimize distortions in the readings of the deformation sensors, ensuring a more accurate assessment of structural conditions. Secondly, the arrangement aimed to reduce thermal interference between the deformation sensors, as variations in ambient temperature can impact their properties. By placing the temperature sensor in the middle, it acted as an indicator of the overall thermal environment, assisting in correcting these variations and contributing to more reliable and consistent measurements of the deformation sensors.

**Figure 1:**
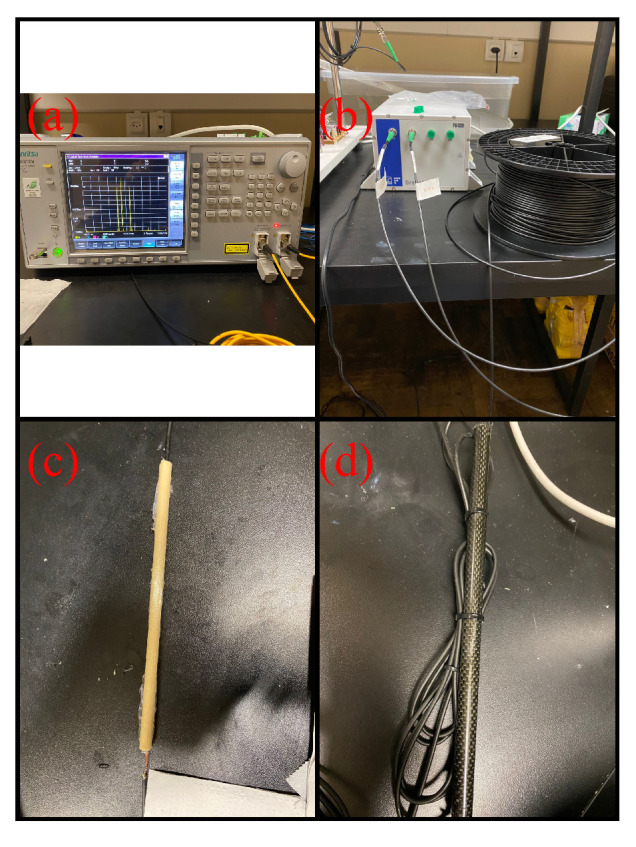
(**a**) Spectral verification of the sensor array; (**b**) interrogation device used in conjunction with the coil and array; (**c**) PLA layer of the connector (inner layer); (**d**) prepared connector.

After configuring the sensors, the array was connected to a 50 m coil to enable the descent of the grid together with the sensor array. It is crucial to highlight that all joints were reinforced with a coating consisting of two layers: the first layer was polylactic acid (PLA), and the second layer was carbon fiber, both internally filled with polyurethane adhesive (PU). This procedure aimed to waterproof the joints, providing additional protection to this fragile region against water-related phenomena. Given the extreme conditions of the river, such as tree trunks, stones, and fish weighing up to 200 kg, a simple thin stainless-steel tube would not suffice to protect the sensors and splices from potential impacts. Therefore, a more robust encapsulation was necessary, consisting of multiple layers designed to withstand such harsh environments. Figure 1 presents (a) the spectral verification of the array; (b) the interrogation device used in conjunction with the coil and array; (c) the PLA layer of the connector (inner layer); and (d) the final designed connector.

### 2.3. Encapsulation Testing

To assess the effectiveness of the encapsulations in preserving sensor sensitivity under real conditions, simultaneous tensile tests were conducted for both deformation and temperature sensors. The purpose of these experiments was to verify whether the deformation sensor would show sensitivity to mechanical tension while the temperature sensor would remain unchanged, i.e., without variation in its Bragg wavelength throughout the experiments.

It is crucial to emphasize that conducting these tests on the sensors intended for the array in a laboratory setting is a challenging task. This is due to the significant distance between the sensors and the array itself, making it impractical to position the sensors appropriately in the calibration deformeter. Therefore, to evaluate the effectiveness of the encapsulations, we utilized sensors with the same characteristics as those intended for the array. Despite the distinct Bragg wavelengths, the sensors exhibited the same calibration curves, as described in Equation (3) for temperature and Equation (7) for deformation.

**Figure 2:**
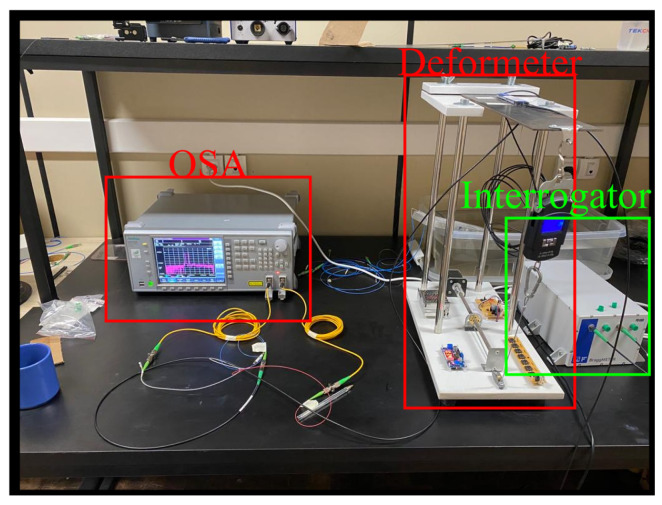
Deformation Test Setup.

For the test, a deformeter was designed to pull a 1040 steel plate. This device comprised a control plate, a stepper motor, a scale, and optically calibrated deformation and temperature sensors serving as references. In the procedure, the operator determined the distance the stepper motor would travel, thereby pulling the plate where the sensor was positioned. The applied tension was measured by both the scale and the reference deformation sensor. Subsequently, the deformation and temperature sensors were placed in parallel with the reference sensors, ensuring that the force applied to them was equivalent to that applied to the deformeter sensors. They were then connected using the spot-welding technique. The test was conducted, initially with no load (0.000 kg), increasing in steps of approximately 0.500 kg up to the maximum load of 2.500 kg. Figure 2 illustrates the execution of the deformation test. Thus, the strain range was from 0 to approximately 350 microstrains. For temperature calibration, the FBG responsible for measuring temperature was placed in a specially adapted oven. The calibration range was from 20 to 70 degrees Celsius.

### 2.4. Experimental Setup

**Figure 3:**
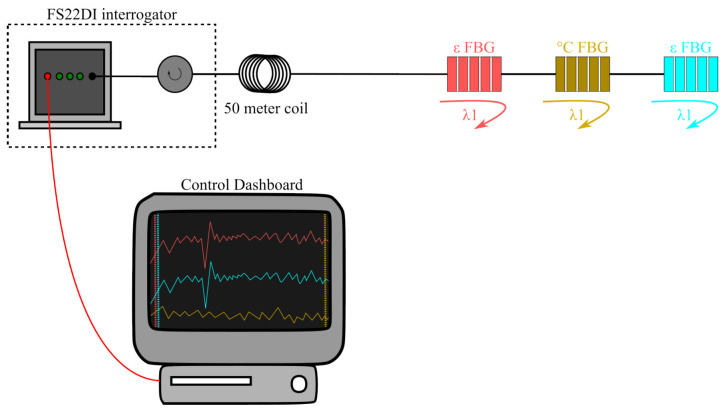
Schematic representation of the setup designed for conducting tests at the hydroelectric power plant.

For the implementation of the sensors in the field, a Braggmeter FS22DI (HBM FiberSensing, Porto, Portugal) was employed to acquire the optical spectrum. Additionally, we developed dedicated software to analyze the collected data related to the deformation and temperature sensors. Figure 3 presents a representation of the setup designed for conducting tests at the hydroelectric power plant. The primary purpose of the control dashboard is to monitor the sensors, allowing verification of the measured strain and temperature levels. Moreover, it serves as preliminary software for the implementation of these arrays in the plant’s monitoring and control room.

To install the array of FBGs on the grid, we employed a combination of epoxy adhesive and spot-welding techniques. Initially, we applied 3M Scotch-Weld Epoxy Adhesive DP460 (3M, Maplewood, MN, USA), a bicomponent structural epoxy adhesive, with a mixing ratio of 2:1, which achieves handling strength in approximately four hours after a 60 min drying time, ensuring a robust initial connection to various surfaces. Subsequently, the FBGs were precisely attached to the metal rods of their encapsulations using spot welding. Spot welding is a specialized method where metal strain gauges are securely welded to metal construction components, utilizing advanced technologies such as the C30 (Walter Heller GmbH, Dieburg, Germany). This technique enhances the reliability and durability of the sensor attachment to the grid structure.

The protective grids, where the sensors were installed, are constructed from 1040 steel, a high-strength carbon steel known for its excellent mechanical properties and durability under varying environmental conditions. The grids were coated with Munsell N-1 (Tigre, Joinville, Brazil) naval-type paint, renowned for its corrosion resistance and adherence to stringent marine and industrial standards

**Figure 4:**
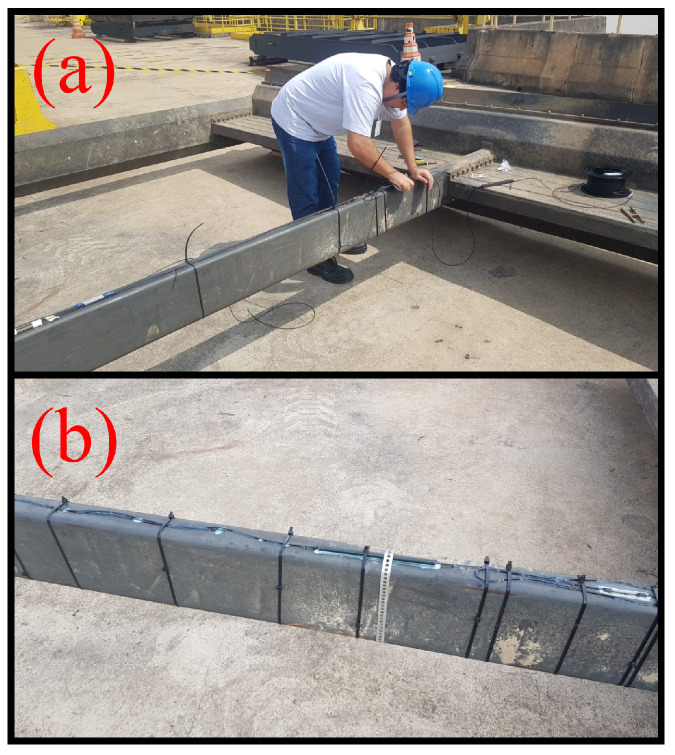
Array installation process on the hydroelectric plant grid: (**a**) installation moment, (**b**) array in place.

After the epoxy adhesive had completely dried, polyurethane (PU) was applied across the entire array for additional protection. Excess cables were secured with cable ties to prevent interference from current flow. Figure 4a depicts the moment of array installation on the grid, showcasing the application of these installation techniques. Figure 4b provides a visual representation of the fully installed array.

## 3. Results

### 3.1. Encapsulation Test Results

**Figure 5:**
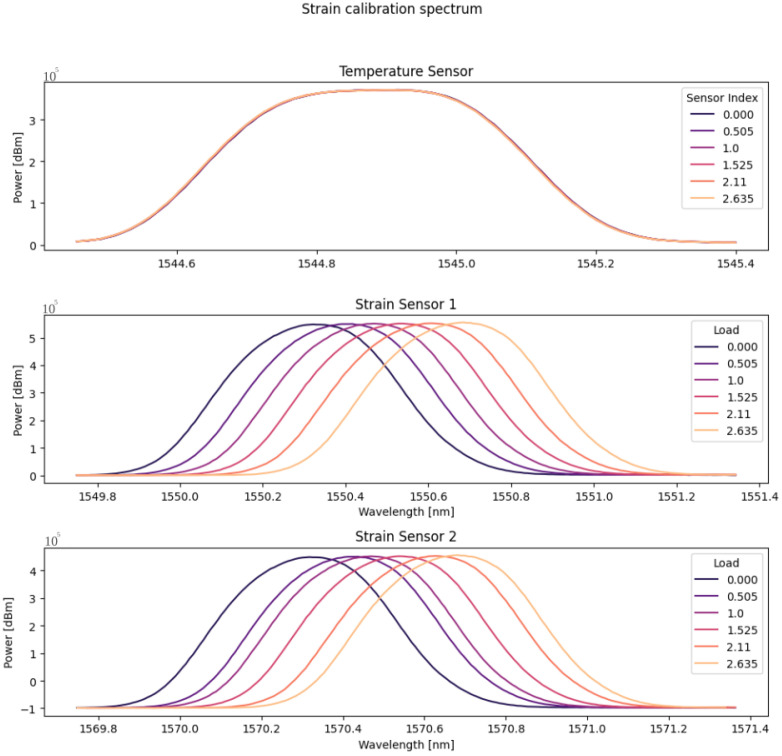
Spectral analysis of the optical sensors during the deformation test.

As a preliminary analysis, spectral verification was initially conducted, as depicted in Figure 5. It was observed that the deformation sensors exhibited variations corresponding to the progressive increase in load, while the temperature sensor remained seemingly inert. It is crucial to note that the conducted test was of short duration, and the laboratory temperature remained constant at 20 °C throughout the entire experiment.

**Figure 6:**
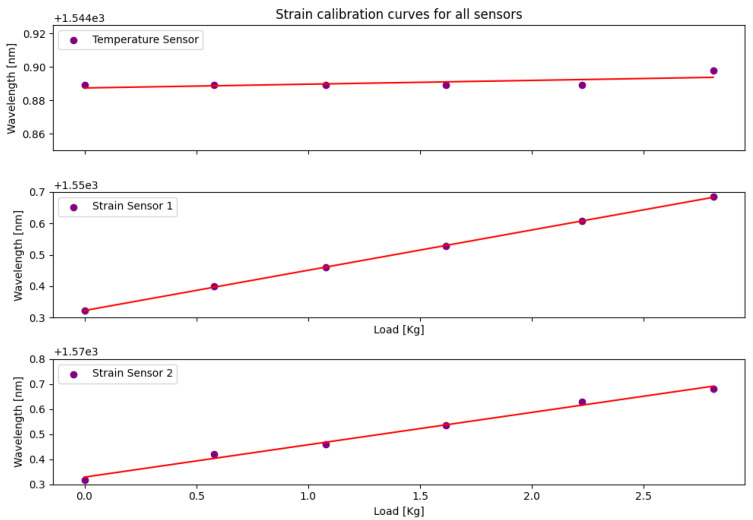
Strain calibration curves for the temperature and strain sensors.

Subsequently, to assess the sensors’ sensitivity more accurately concerning deformation, calibration curves for the utilized devices were acquired. Figure 6 illustrates the calibration curves for the temperature and strain sensors, respectively. In the figure, it is evident that the temperature sensor exhibited a sensitivity close to 0, while the deformation sensors demonstrated sensitivity to load variations.

Upon analyzing Equations (8)–(10), the sensitivity to deformation in the temperature sensor and the strain sensors becomes evident. It is crucial to note that in these equations, $\lambda_{b}$ represents the Bragg wavelength, and *x* denotes the applied load.

(8) $$\lambda_{b_{temp}}=0.00226\cdot \mathrm{Load}+1544.88746$$

(9) $$\lambda_{b_{strain1}}=0.12854\cdot \mathrm{Load}+1550.32209$$

(10) $$\lambda_{b_{strain2}}=0.12907\cdot \mathrm{Load}+1570.32871$$

Based on the statistical analysis of each sensor, the temperature sensor exhibited a coefficient of determination ($R^{2}$) of 0.44724, indicating a moderate correlation between the temperature and wavelength shift. Its sensitivity to strain was calculated at 0.00226 nm/kg, reflecting a minimal response to load variations.

The strain sensors showed significantly higher coefficients of determination. Strain Sensor 1 achieved an $R^{2}$ of 0.99986, demonstrating a strong correlation between the applied load and Bragg wavelength shift, with a sensitivity of 0.12854 nm/kg. Similarly, Strain Sensor 2 obtained an $R^{2}$ of 0.99219 and a slightly higher sensitivity of 0.12907 nm/kg.

**Figure 7:**
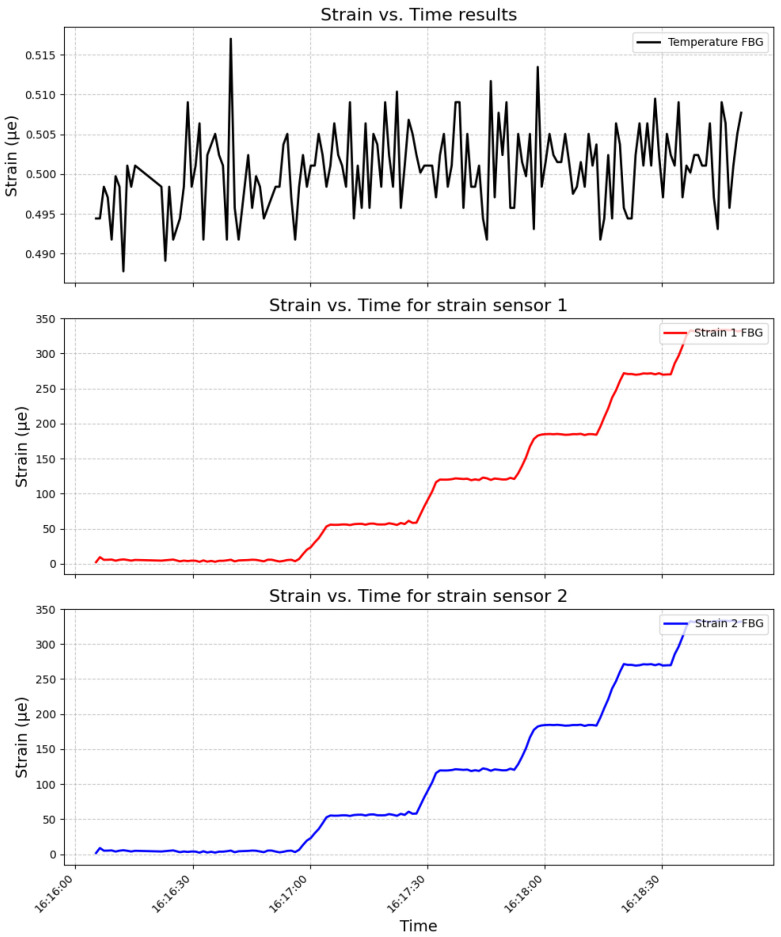
Relationship between applied load and time for the temperature sensor and two strain sensors, illustrating their sensitivity to deformation.

Figure 7 depicts the deformation data obtained for each sensor throughout the experiment. The results indicate significant differences in strain sensitivity between the temperature sensor and the two strain sensors, as evidenced by their respective calibration curves and statistical analyses.

**Figure 8:**
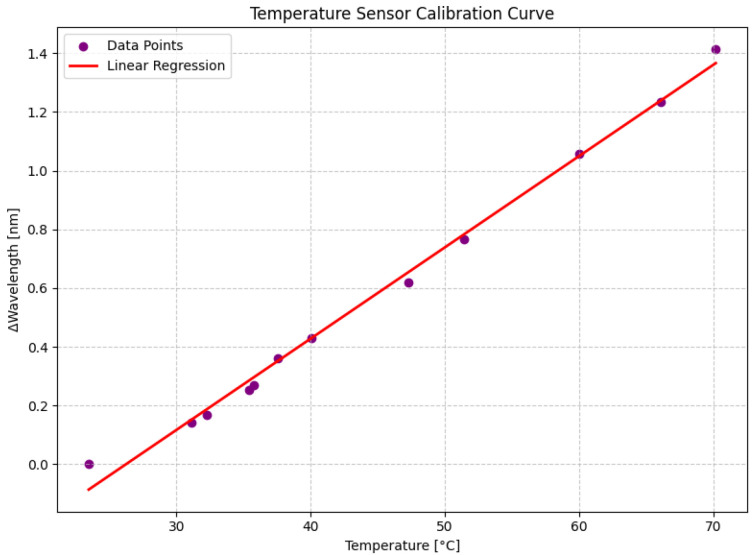
Calibration curves for temperature.

To accurately assess the sensitivity of the temperature sensor to temperature variations, calibration curves were obtained for the device. Figure 8 displays the calibration curve for the temperature sensor, illustrating the relationship between temperature changes and the corresponding wavelength shifts. The equation of the calibration curve is given by:

(11) Δλ=0.03109·Temperature−0.81580

where Δλ represents the wavelength shift in nanometers and Temperature represents the temperature in degrees Celsius. The high coefficient of determination ($R^{2}=0.99417$) indicates a strong correlation between the temperature and wavelength shift, validating the sensor’s accuracy. The sensitivity of the temperature sensor to temperature changes was calculated as 0.03109 nm/°C, highlighting its precision in temperature measurement.

The designed sensors’ performance indicators were thoroughly evaluated through various tests. The encapsulation effectiveness was assessed by subjecting both the deformation and temperature sensors to simultaneous tensile tests under real conditions. The deformation sensors demonstrated sensitivity to mechanical tension, exhibiting variations corresponding to the applied load, while the temperature sensor showed stability, with no significant changes in its Bragg wavelength throughout the experiments. Calibration curves further confirmed the deformation sensors’ responsiveness to load variations and the temperature sensor’s accuracy, maintaining a sensitivity close to zero for deformation and high precision in temperature measurement. These results underscore the robustness and reliability of the sensor design in maintaining sensitivity and accuracy under field conditions.

### 3.2. Field Test Results

**Figure 9:**
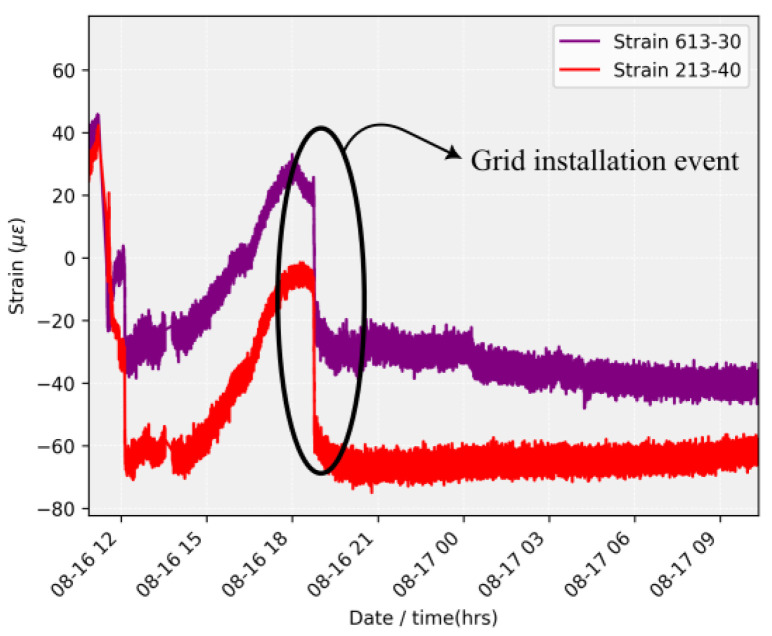
Strain history capturing the descent.

After the installation of the sensors, two significant events were observed. The first event occurred shortly after the array installation during the descent of the protective grid. Figure 9 illustrates the time series of the strain sensors during the maneuver to position the grid. In this image, it is possible to identify an event on 16 August at 18:00 h, precisely marking the descent of the grids and the contact of the sensors with the water.

**Figure 10:**
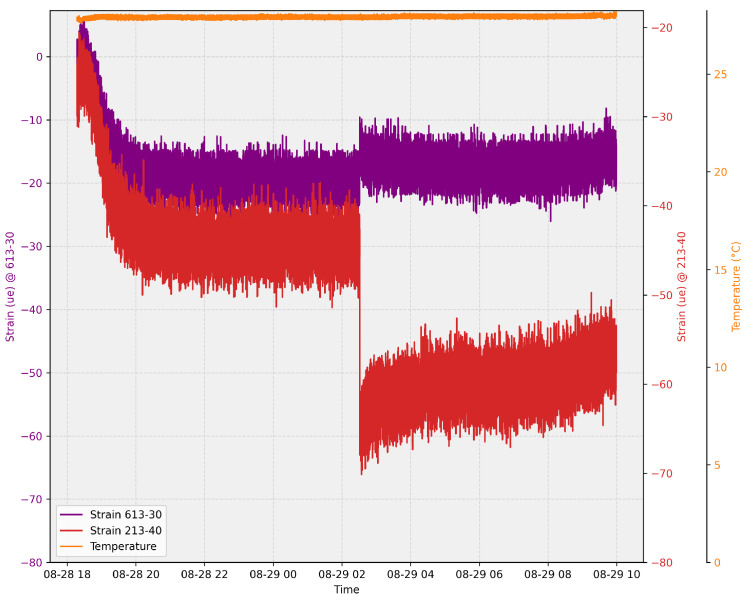
Time series of sensors during the macrophyte event.

**Figure 11:**
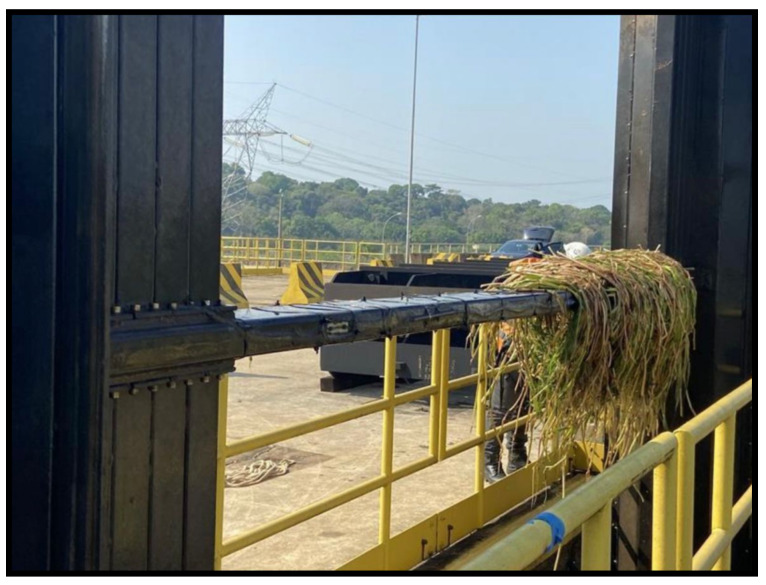
Macrophyte encounter: optical image.

The second significant event occurred on 29 August at 2:00 AM, where a significant deformation was observed in one of the sensors, which remained constant after the incident. This alerted the power plant engineers, who promptly initiated the maintenance process. Upon lifting the grids, a cluster of macrophytes was found to have collided with the grid. Figure 10 depicts the temporal series of the sensors during this event, and Figure 11 showcases the cluster of macrophytes encountered.

## 4. Discussion

The field test results demonstrated the high performance of the designed sensor array in real-time monitoring of underwater structures. Specifically, two significant events highlight the sensors’ capabilities: the descent of the protective grid and an encounter with macrophytes. During the grid descent, the strain sensors accurately captured the deformation caused by the maneuver, showcasing their sensitivity to mechanical changes. The temperature sensors, however, showed minimal response, indicating their stability and specificity to temperature variations.

On 29 August, a significant deformation was detected by one of the strain sensors at 2:00 a.m., which remained constant after the incident. This event triggered an immediate maintenance response from the power plant engineers, leading to the discovery of a cluster of macrophytes that had collided with the grid. The prompt detection and alert system underscores the effectiveness of the sensor array in enhancing the maintenance and safety of underwater structures. The high sensitivity of the strain sensors ($R^{2}$ = 0.99986 for Strain Sensor 1, and $R^{2}$ = 0.99219 for Strain Sensor 2) and the precise temperature measurement capability (sensitivity of 0.03109 nm/°C) validate the robustness and accuracy of the proposed monitoring system.

The encapsulation test results of our sensors demonstrate their high sensitivity and stability. The deformation sensor showed significant responsiveness to mechanical tension, accurately reflecting variations corresponding to the applied load. Similarly, the temperature sensor maintained its precision, with negligible changes in the Bragg wavelength during the tests, indicating high stability and reliability under field conditions.

In contrast, the study by [42] provided a comprehensive review of various Fiber Bragg Grating (FBG)-based temperature and strain sensors. The reviewed sensors exhibited a broad range of sensitivities and accuracies depending on their design and application context. For instance, the FBG-based sensors showed high sensitivity to temperature changes and strain, making them suitable for applications in harsh environments, structural health monitoring, and various industrial sectors.

Our sensors’ performance is comparable to the best-performing sensors discussed in the literature, particularly in terms of stability and sensitivity. However, unlike some advanced FBG designs, such as secondary gratings and ROGUE gratings, which offer enhanced performance metrics and broader applicability, our design focuses on achieving high reliability and accuracy within specific operational conditions.

This comparison underscores the robustness of our sensors in maintaining accurate readings under practical conditions while highlighting the potential for future enhancements by integrating advanced FBG techniques to further improve performance metrics such as sensitivity and operational range.

## 5. Conclusions

The research presented in this study demonstrates that the use of FOSs as an approach for real-time monitoring of underwater structures is highly efficient in identifying and responding to critical events in aquatic environments. Through laboratory tests, including encapsulation and calibration trials, the sensitivity and accuracy of the sensors in field conditions were ensured. The results highlight the deformation sensor’s consistent response to load variations, while the temperature sensor remained inert, affirming the robustness of the proposed monitoring system.

Field tests further validated the effectiveness of the sensor array in detecting significant events, such as the descent of protective grids and encounters with macrophytes. The sensors’ real-time detection and recording of these events underscore the system’s potential for enhancing the maintenance and safety of underwater structures. While this study provides promising results, continuous improvements, including expanding the sensor array and integrating advanced data analysis algorithms, are recognized as avenues for future development.

In conclusion, the use of fiber-optic sensors for underwater monitoring holds tremendous potential, not only for the specific case discussed but also for advancing structural health monitoring (SHM) strategies in hydroelectric plants and similar critical infrastructure.

## Data Availability

The data that have been used are confidential.
